# Critical role of guanylate binding protein 5 in tumor immune microenvironment and predictive value of immunotherapy response

**DOI:** 10.3389/fgene.2022.984615

**Published:** 2022-09-30

**Authors:** Xiang Li, Dan Song, Song Su, Xiaobo He, Fengyu Cao, Chao Yang, Kai Li, Shuoyang Huang, Changhua Li, Chenhong Wang, Aikang Zhang, Pengcheng Pang, Yongbin Zheng

**Affiliations:** ^1^ Department of Gastrointestinal Surgery, Renmin Hospital of Wuhan University, Wuhan, China; ^2^ The Fifth Medical Center of Chinese PLA General Hospital, Beijing, China

**Keywords:** guanylate binding protein 5, pan-cancer, colorectal cancer, immunotherapy, tumor immune microenvironment, prognosis

## Abstract

**Background:** The guanylate-binding proteins (GBPs) are the latest potential targets of immunotherapy. However, the role of GBP5 in pan-cancer, including colorectal cancer (CRC), remains unclear. This study aims to explore the effect of GBP5 on immunity in pan-cancer.

**Methods:** Based on the RNA sequencing data of 33 cancers obtained from The Cancer Genome Atlas, we analyzed the clinical significance of GBPs and focused on the correlation between GBP5 and tumor microenvironment (TME). Immunotherapy cohort IMvigor210 was used to explore the relationship between treatment response and GBPs. Then, we further analyzed the expression of GBP5 in immune cells using single-cell transcriptome cohort GSE146771 and GSE132465 from the Gene Expression Omnibus database. Finally, a prognostic model based on GBP5 expression was established and validated.

**Results:** We found that the expression of GBP3/4/5 is higher in colorectal cancer than in normal tissues, and GBP5 is a better predictor of good treatment response to immune checkpoint blockade than other GBPs. In most other cancers, GBP5 is also elevated in tumors compared with normal tissues and is associated with a better prognosis. As for TME, GBP5 is generally positively correlated with immune score, the level of tumor-infiltrating immune cells and immune-related genes. Single-cell analysis showed that GBP5 was mainly expressed in myeloid cells and T cells. The GBP5-related prognostic model we constructed in CRC can predict the survival of patients and propose some genes for subsequent research.

**Conclusion:** This study revealed a strong correlation between GBP5 and immunity in generalized cancer and provided evidence that CRC may be a suitable cancer type for anti-GBP5 therapy.

## Introduction

Cancer is an ongoing public health problem throughout the world, with colorectal cancer (CRC) being one of the most common cancers. In the United States, there were an estimated 147,950 new cases and 53,200 deaths according to recent cancer statistics (Siegel et al., 2020). In these years, while the use of colorectal cancer screening tests has led to a decline in CRC death rates, the latest studies report that CRC death rates are increasing among adolescents and young adults (Henley et al., 2020). At present, tumor immunotherapy represented by immune checkpoint blockade (ICB) (such as PD-L1, PD-1, CTLA-4 blockade) has made progress in some cancers (Rosenberg et al., 2016). In addition to tumor cells, cells in solid malignant tumors also include vascular endothelial cells, fibroblast cells, and a variety of innate and adaptive immune cells (Duan et al., 2020). Immune cells in the tumor microenvironment (TME) are composed not only of immunosuppressive cells but also of tumor-fighting effector cells. TME can be classified as cold or hot due to the production of pro-inflammatory cytokines and infiltration of T cells. Previous studies have shown that ICB efficiency is closely related to TME (Gajewski et al., 2017). For example, hot tumors have a high rate of response to ICB treatment. Therefore, it is of great significance to accurately describe TME and find the key molecules.

The guanylate-binding proteins (GBPs) are members of the TRAFAC class dynamin-like GTPase superfamily (Vestal and Jeyaratnam, 2011). Humans are thought to have seven GBPs, known as GBP1-7. As one of the GBPs, GBP5 has a variety of biological functions. In tumors, it has been suggested that higher GBP5 mRNA expression levels can predict a good prognosis of triple-negative breast cancer, but the mechanism remains unclear (Cheng et al., 2021a). GBP5 is also associated with pyroptosis, which is a phenotype that plays a crucial role in tumors and is prevalent in immune responses. Inflammasomes including NLRP3 and AIM2 can induce pyroptosis. GBP5 is involved in regulating NLRP3 activation by pathogenic bacteria and soluble but not crystalline inflammasome priming agents (Shenoy et al., 2012). Similarly, GBP5 is the key activator protein of AIM2 which activates caspase-1 in mouse macrophages through ASC in response to Francisella infection (Meunier et al., 2015). In addition, GBP5 has been shown to enhance tumor immunogenicity and has the potential to be a target for cancer immunotherapy (Li et al., 2021). Nevertheless, the role of GBP5 in most tumors, especially CRC, remains to be further studied.

In this study, we analyzed the relationship between GBP5 and clinical indicators or TME in pan-cancer. We also explored the correlation between GBP5 and immunotherapy and found that CRC may be an appropriate tumor type to target GBP5. In addition, we performed a single-cell analysis and constructed a GBP5 associated prognostic model in CRC. Overall, our study reveals the relationship between GBP5 and tumor immunity and provides evidence for targeted GBP5 therapy.

## Materials and methods

### Data sources

Pan-cancer RNA-seq and corresponding clinical data and somatic mutation data in The Cancer Genome Atlas (TCGA) were downloaded from UCSC Xena datasets (Goldman et al., 2020). Proteome data for colorectal cancer and corresponding normal tissues were obtained from the CPTAC database. Immunotherapy cohort IMvigor210 was downloaded from a previous study, which was a cohort of bladder urothelial carcinoma using atezolizumab intervention (Mariathasan et al., 2018). In addition, colon cancer cohort GSE17537, single-cell transcriptome cohort GSE146771 and GSE132465 were obtained from the Gene Expression Omnibus (GEO) database.

### Effect of GBPs on immunotherapy response

To begin with, we analyzed the clinical cohort IMvigor210 using PD-L1 inhibitors to determine the relationship between GBPs and immunotherapy. We compared GBPs expression levels in four groups of patients who responded differently to ICB: progressive disease (PD), stable disease (SD), partial response (PR), and complete response (CR). We identified 13 immunotherapy-positive signaling pathways from the Kyoto Encyclopedia of Genes and Genomes (KEGG) database and calculated enrichment scores for each pathway in CRC using the “GSVA” R package (Hänzelmann et al., 2013; Hu et al., 2021). Then, we compared the enrichment scores of the high and low GBP5 expression groups in CRC.

### Clinical correlation analysis in pan-cancer

GEPIA2 was used to analyze whether GBPs expression in tumor or normal samples in TCGA and GTEx was significantly different (Tang et al., 2019). We also divided the patients into GBP5 high and low expression groups according to the cut-off value and used the Kaplan-Meier method to analyze the prognosis based on the overall survival. In addition, we compared the correlation between GBP5 expression and various clinical indicators, including gender, age, and TNM stage. *p* < 0.05 is considered significant difference.

### Evaluation of the correlation between GBP3/4/5 and TME in pan-cancer

To understand the infiltration level of tumor-infiltrating immune cells (TIICs), we calculated the stromal score, immune score, and tumor purity in each sample using the ESTIMATE package in R and correlated them with the corresponding GBP3/4/5 expression. ESTIMATE is an algorithm based on ssGSEA for estimating the abundance of stromal, immune, or malignant cells in tumor tissue (Yoshihara et al., 2013). To further estimate the infiltration level of TIICs, CIBERSORT and ssGSEA were used to calculate the infiltration level of each immune cell. CIBERSORT is a deconvolution algorithm that calculates the abundance of 22 immune cell subsets in all immune cells (Newman et al., 2015). Similarly, ssGSEA was used to calculate the enrichment scores of 28 immune cells in each sample (Hänzelmann et al., 2013). Finally, we obtained immune-related genes including immune inhibitors, immune stimulators, and MHC molecules from previous studies and compared their correlation with GBP3/4/5 expression (Charoentong et al., 2017).

### GBP5 immunogenicity analysis

We further analyzed some factors related to immunotherapy effect or immunogenicity, such as tumor mutation burden (TMB) and microsatellite instability (MSI). TMB was calculated by VarScan2 from somatic mutation data from TCGA and MSI was obtained from a previous study (Bonneville et al., 2017). In addition, we defined the CD8A expression in the top 10% of CRC as hot tumors and the bottom 10% as cold tumors. Hot tumors were associated with a higher rate of response to ICB therapy and CD8A reflected the degree of T cell infiltration. The expression differences of GBP5 in cold and hot tumors were then compared.

### Single-cell RNA-seq analysis in CRC

A total of 57,979 CRC cells were included in two cohorts for analysis. We used the “Seurat” R package for quality control and statistical analysis (Butler et al., 2018). After the exclusion of low-quality genes and cells, PCA and UMAP methods were successively used for dimensionality reduction. We ended up with 20 clusters. Each cell cluster was annotated using the “SingleR” package and manually validated with the CellMarker database (Aran et al., 2019; Zhang et al., 2019a). Finally, we compared the mRNA expression of GBP5 and some immune-related genes at the single-cell level.

### Biological function analysis of GBP5

CRC patients were divided into GBP5 high and low expression groups. Then, we performed gene set enrichment analysis (GSEA) to identify functions and signaling pathways. The functions and pathways were derived from the Gene Ontology (GO) and KEGG databases. The criteria for presented results were |NES| > 2, p-val < 0.01, and FDR q-val < 0.05. Next, we used the Wilcox test to identify differentially expressed genes (DEGs) from two groups. DEGs with FDR filter <0.05 and logFC filter >1 were obtained. Then, GO and KEGG analysis was performed and pathways with the highest enrichment were selected for display.

### Development and verification of GBP5 related risk score

First, survival-related DEGs were obtained by univariate Cox analysis. Next, we randomly divided CRC samples in TCGA into training sets and test sets in a ratio of 6:4. A survival-related risk score was obtained using multivariate Cox regression. We assessed the correlation between risk score and survival using the Kaplan-Meier method and evaluated its statistical performance using ROC curves. At the same time, we use the test set to verify.

### Cell culture and construction of lentiviral cell lines

We purchased the human colorectal cancer cell line DLD1 from Procell Life Science& Technology Co., Ltd (Wuhan, China). Cells were maintained in RPMI-1640 supplemented with 10% FBS and incubated at 37 °C in a humidified atmosphere of 5% CO_2_. Cells were transfected with overexpression and knockdown lentiviral vectors designed and supplied by HANBIO Co., Ltd. (Shanghai, China). GBP5 knockdown shRNA sequence was as follows: GAT​GAT​GAG​CTA​GAG​CCT​GAA. The transfection MOI was approximately 50. Cells were treated with 4 μg/ml puromycin for 2 weeks at 72 h after infection.

### Western blot analysis

We obtained total proteins using RIPA buffer containing Millipore protease inhibitors. Proteins were separated on SDS-PAGE gels and then blotted onto nitrocellulose membranes. Nonspecific binding was blocked with TBST containing 5% milk for 1–2 h. We incubated the primary antibody with the membrane overnight at 4°C. The antibodies used were GBP5 antibodies (Cell Signaling, United States, cat#67798, 1:1,000) and tubulin antibodies (Proteintech, Wuhan, China, cat#11224‒1-AP, 1:20,000). Next, membranes were rinsed 2–3 times with TBST and inoculated with HRP-labelled secondary antibodies. Finally, visualization was performed with the Bio-Rad ChemiDoc XRS + imaging system.

### 5-ethynyl-20-deoxyuridine incorporation assay and colony formation assay

We cultured DLD1 cells in 96-well plates. At 37 °C, cells were treated with 50 μM 5-ethynyl-20-deoxyuridine (EdU, Ribobio, China) for 2 h. Then, the cells were fixed with formaldehyde for 30 min and treated with 0.5% Triton X-100 for 10 min. Next, 100 μl of 1 × Apollo^®^ reaction cocktail was added to each well for 30 min. Finally, we stained the cells with 100 μl of 1 × Hoechst 33,342 for 30 min and observed them under a fluorescence microscope. We seeded DLD1 cells at a density of 1,000 cells per well on 6-well plates for colony formation assays. The cells were cultured for 2 weeks, during which the culture medium was changed once every 3 days. At last, the number of the colony in each well was stained and counted.

### Statistical analysis

We used the Pearson coefficient to explore the correlation between variables. One-way ANOVA or Wilcoxon test was used to compare differences between groups. Kaplan-Meier method was used to plot survival curves for prognostic analysis. A *p*-value < 0.05 was considered statistically significant. We used 4.1.1 version R software for statistical data analysis.

## Results

### Differential expression of GBPs and prediction of ICB response

**FIGURE 1:**
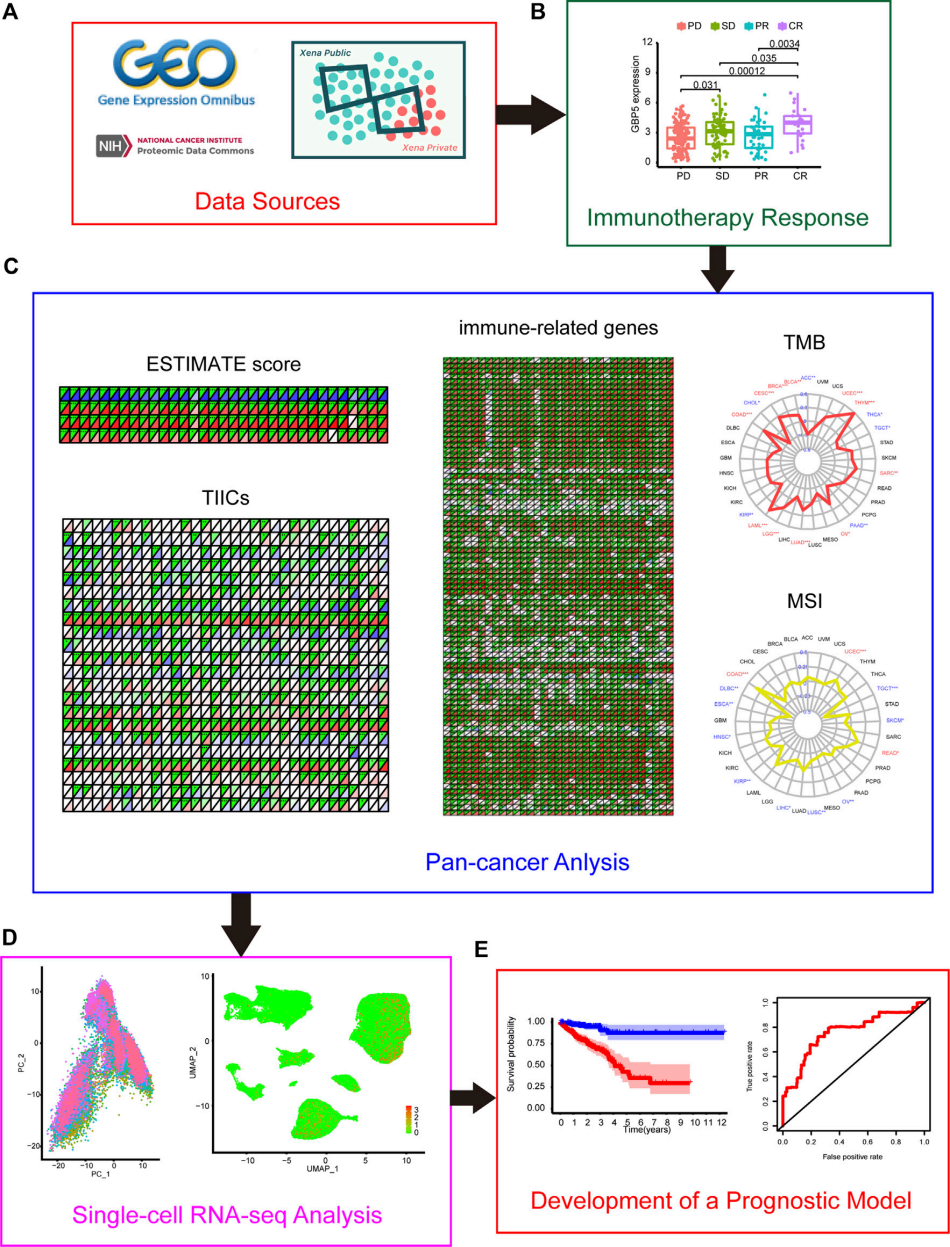
Flow chart of this study **(A)** Data sources of this work. **(B)** The correlation between GBPs (guanylate-binding proteins) and the effect of immunotherapy **(C)** Correlation between GBP5 and TME (tumor microenvironment) in pan-cancer **(D)** Single-cell analysis of GBP5 in CRC (colorectal cancer). **(E)** Construction of a GBP5-related prognostic model in CRC.

**FIGURE 2:**
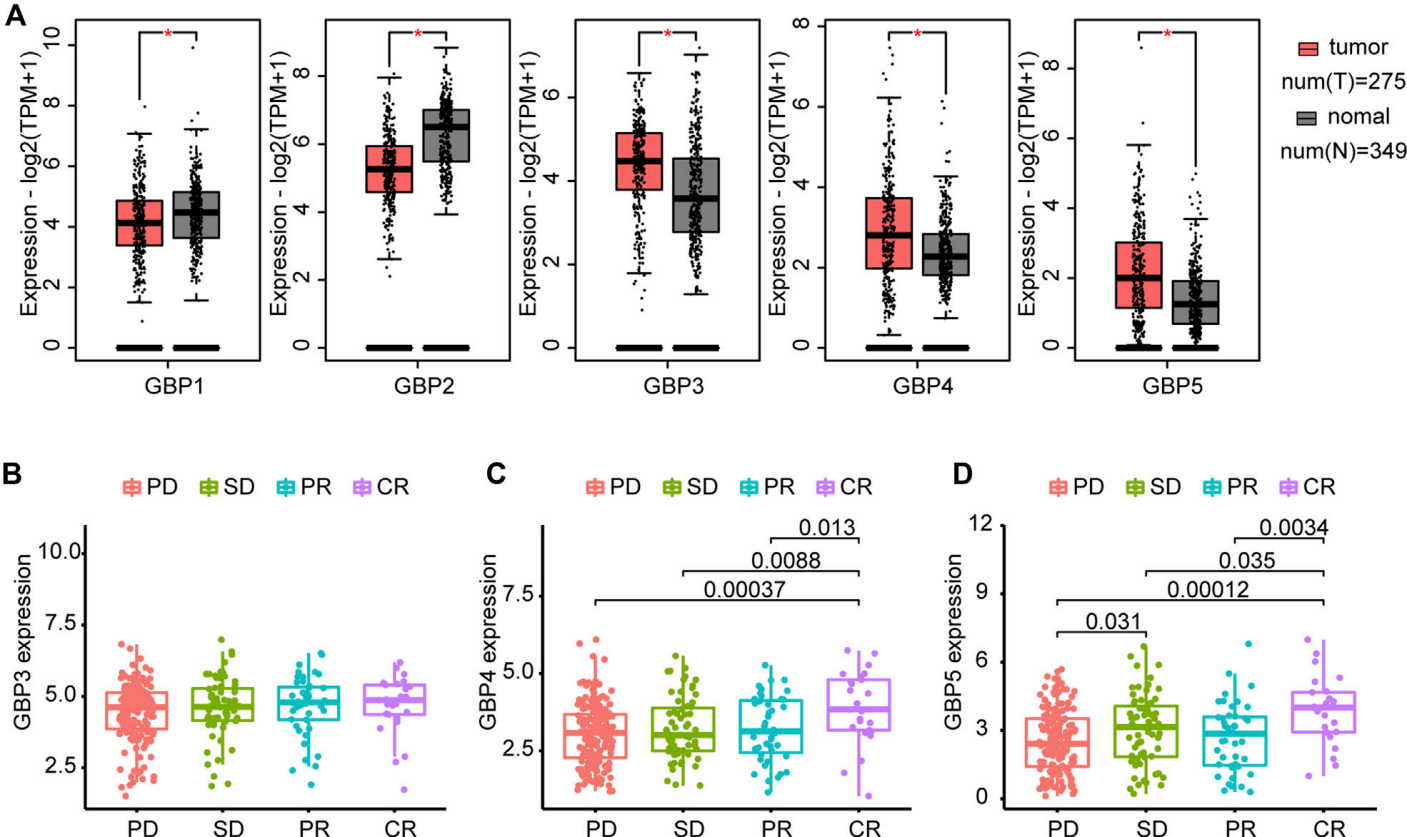
Differential expression of GBPs and prediction of ICB (immune checkpoint blockade) response **(A)** GBPs expression difference between tumor and normal tissues in COAD (Colon adenocarcinoma) by GEPIA2. The expressions of GBP1 and GBP2 in normal tissues are significantly higher than those in tumor tissues. The expressions of GBP3, GBP4, and GBP5 in tumor tissues are significantly higher than those in normal tissues. “*” indicates *p* < 0.05 **(B)** Differences of GBP3 expression between groups with different responses to immunotherapy in the IMvigor210 cohort. GBP3 expression is 4.451806 ± 1.031910 in PD group, 4.613511 ± 1.052200 in SD group, 4.669342 ± 1.065907 in PR group and 4.679646 ± 1.037475 in CR group. **(C)** Differences of GBP4 expression between groups with different responses to immunotherapy in the IMvigor210 cohort. GBP4 expression is 3.039644 ± 0.9899737 in PD group, 3.232032 ± 1.0081706 in SD group, 3.205698 ± 1.0478816 in PR group and 3.886223 ± 1.1831482 in CR group. **(D)** Differences of GBP5 expression between groups with different responses to immunotherapy in the IMvigor210 cohort. GBP5 expression is 2.544213 ± 1.364970 in PD group, 3.044128 ± 1.554900 in SD group, 2.697051 ± 1.501733 in PR group and 3.842866 ± 1.478431 in CR group. PD: progressed disease; SD: stable disease; PR: partial response; CR: complete response.

The flowchart shown in Figure 1 summarizes the overall process of this study. 33 tumor full names and abbreviations in TCGA are listed in Supplementary Table S1. By analyzing the mRNA expression of GBPs in pan-cancer, we found that GBP1-5 were highly expressed in tumor tissues, while GBP6 and GBP7 were less expressed (Supplementary Figure S1). We also analyzed the expression of GBP1-5 in tumor tissues and normal tissues in COAD (Colon adenocarcinoma) using GEPIA2 (Figure 2A). The results showed that the expressions of GBP1 and GBP2 in normal tissues were significantly higher than those in tumor tissues. On the contrary, the expressions of GBP3, GBP4, and GBP5 in tumor tissues were significantly higher than those in normal tissues. In addition, we analyzed the differential expression of GBP5 in pan-cancer and found that in most cancer types (22/31), GBP5 expression in tumor tissues was higher than that in normal tissues (Supplementary Figure S2). We also analyzed the CPTAC proteome database using UALCAN (Supplementary Figure S3). At the protein level, GBP5 expression was also higher in colorectal tumor tissues than in normal tissues (*p* = 5.20438594686653e-07). As for the rest of the GBPs, contrary to RNAseq data, GBP1 expression was higher in tumors than in normal tissues (*p* = 1.460226e-04). Consistent with RNAseq data, GBP2 expression was higher in normal tissues (*p* = 1.877130e-04), and GBP4 expression was higher in tumor tissues (*p* = 1.25852508986603e-08).

Furthermore, we compared GBP3, GBP4 and GBP5 expression levels in patients who had different responses to anti-PD-L1 treatment in the IMvigor210 cohort (Figures 2B–D). The result indicated no significant difference in the expression of GBP3 in patients with different responses. GBP4 and GBP5 were significantly elevated in patients with complete response to ICB. However, unlike GBP5, GBP4 showed no significant difference in patients with progressed disease and stable disease. Combined with the above results, GBP3/4/5 are highly expressed genes in tumors, but GBP5 has the strongest ability to predict ICB response. In addition, GBP5 was positively correlated with the enrichment scores of immunotherapy-positive gene sets (Supplementary Figure S4). Therefore, we think that GBP5 is the most valuable and immune-related gene in GBPs.

### Clinical characteristics of GBP5 expression in pan-cancer

**FIGURE 3:**
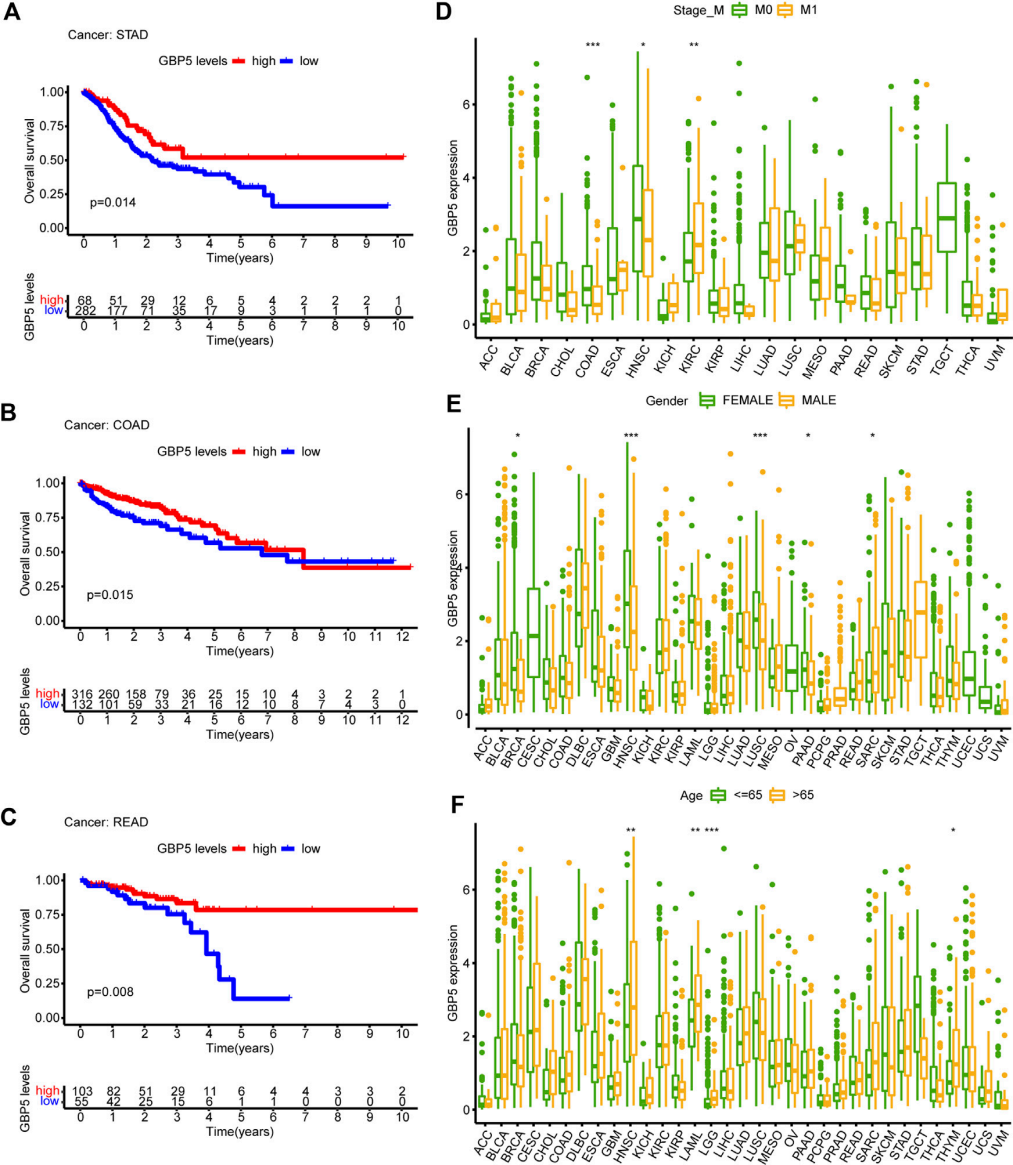
Clinical characteristics of GBP5 in pan-cancer **(A–C)** In STAD (Stomach adenocarcinoma), COAD (Colon adenocarcinoma), and READ (Rectum adenocarcinoma), the samples are divided into two groups according to the optimum cut-off value. Kaplan-Meier curves show that the high GBP5 expression group had a better short-term prognosis than the low GBP5 expression group. **(D–F)** Differences of GBP5 expression in different M stage groups, sexgroups, and age groups. The Wilcoxon test is used for the significance test. “*” indicates *p* < 0.05, “**” indicates *p* < 0.01 and “***” indicates *p* < 0.001.

The elevated expression level of GBP5 in tumors makes it necessary to analyze its correlation with the prognosis of patients. Therefore, we performed a Kaplan-Meier analysis using overall survival in pan-cancer and found that GBP5 was associated with a better short-term prognosis in a majority of cancers such as STAD (Stomach adenocarcinoma), COAD, and READ (Rectum adenocarcinoma) (Figures 3A–C, Supplementary Figure S5). Meanwhile, the expression of GBP5 was also associated with M stage of tumor TNM stage in COAD (Figure 3D), suggesting that GBP5 may influence CRC metastasis. In addition, gender and age were found to have little relationship with GBP5 expression (Figures 3E,F). In summary, although it is not closely related to some clinical parameters, GBP5 has the potential to be a prognostic biomarker for a variety of cancers.

### Correlation between GBP5 and TME

**FIGURE 4:**
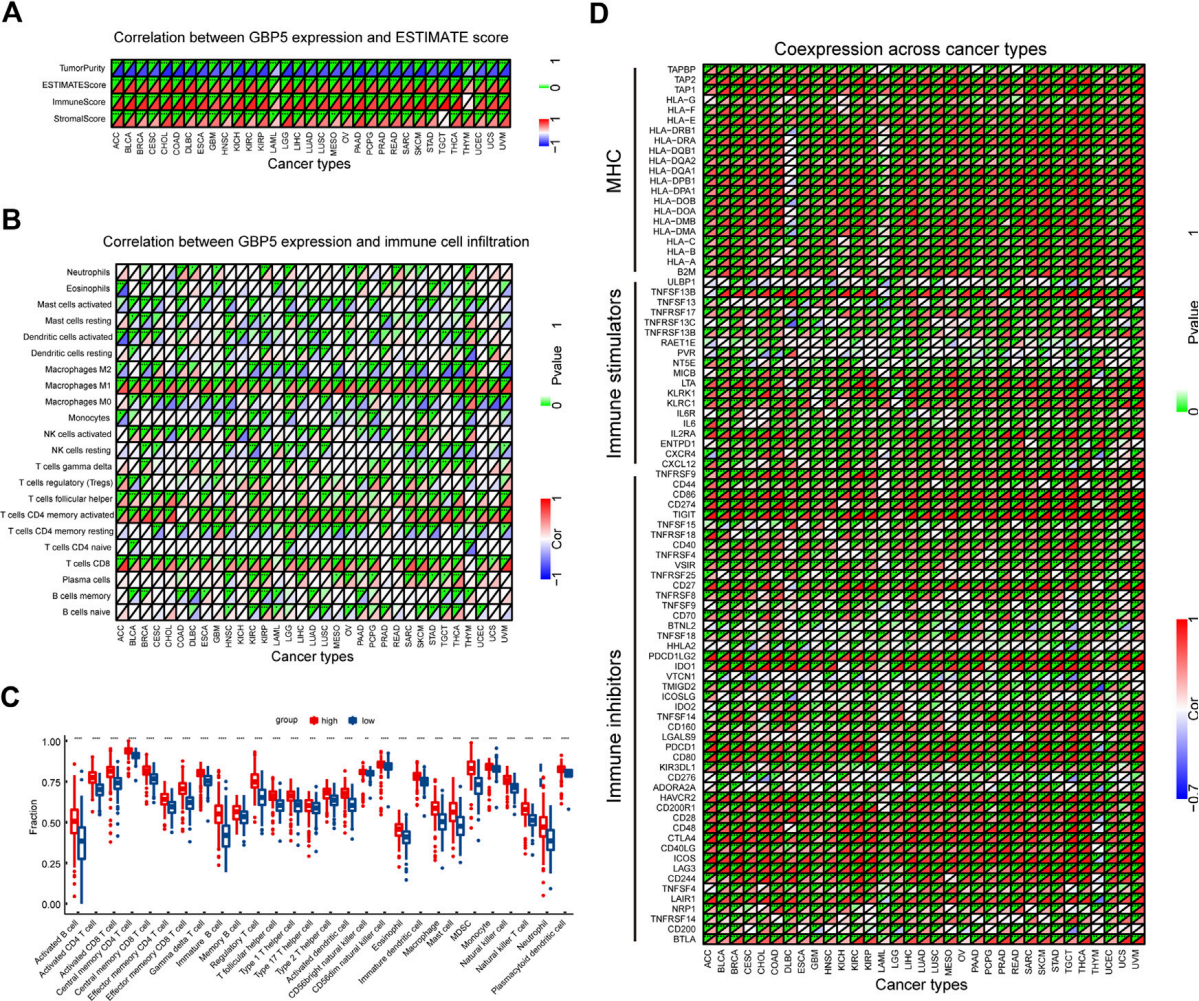
Correlation between GBP5 and TME in pan-cancer **(A)** Correlation between GBP5 and ESTIMATE score. **(B)** Correlation between GBP5 and the infiltration level of TIICs (tumor-infiltrating immune cells) by CIBERSORT **(C)** Differences of the fraction of TIICs by GSEA (gene set enrichment analysis) between GBP5 high and low expression groups in CRC **(D)** The expression correlation between GBP5 and immune-related genes. Sorted by correlation values in COAD, the top ten genes were PDCD1LG2 (R = 0.881840593), CD274 (R = 0.865101814), CD86 (R = 0.84128397), TIGIT (R = 0.838645959), HAVCR2 (R = 0.815956551), TNFRSF9 (R = 0.813663547), LAIR1 (R = 0.788751933), CD80 (R = 0.787116939), ICOS (R = 0.767427362), and CTLA4 (R = 0.760401416). “*” indicates *p* < 0.05, “**” indicates *p* < 0.01 and “***” indicates *p* < 0.001.

In order to determine the immunological effects of GBP5 and find potential tumor types suitable for anti-GBP5 immunotherapy, a correlation between GBP5 expression with immune score, stromal score, and tumor purity was demonstrated (Figure 4A). We found that GBP5 was positively correlated with immune score and stromal score, but negatively correlated with tumor purity in almost all tumors, which was validated in an additional cohort (Supplementary Figure S6). We also used ssGSEA or CIBERSORT method to obtain the infiltration of immune cells in CRC or pan-cancer and compared its correlation with GBP5 expression (Figures 4B,C). The results showed that GBP5 expression was positively correlated with the abundance of all the immune cells by ssGSEA. In particular, there was a strong, positive correlation between GBP5 expression with the abundance of Macrophages M1, T cells follicular helper, T cells CD4 memory activated, and T cells CD8 using CIBORSERT. Furthermore, we analyzed the RNA expression correlation of immune inhibitors, immune stimulators and MHC with GBP5 (Figure 4D). We found that many immune-related genes were positively correlated with GBP5. Among them, the top ten correlated genes are immune inhibitors, and their upregulation is one of the key features of an inflamed TME. Other immune inhibitors positively correlated with GBP5 included some clinically common immunotherapy targets, such as PD-1 (R = 0.676,497,252, *p* = 2.65e-64 in COAD), PD-L1 (R = 0.865101814, *p* = 1.24e-142 in COAD), and CTLA-4 (R = 0.760401416, *p* = 5.88e-90 in COAD), suggesting the potential of GBP5 in clinical application. In addition, we analyzed the correlation of GBP3 and GBP4 with TME and obtained similar results for GBP5 (Supplementary Figure S7, S8). We also analyzed the correlation between GBP5 and inflammasome NLRP3 or AIM2, which has been reported in other diseases (Shenoy et al., 2012; Meunier et al., 2015) (Supplementary Figure S9). As we expected, NLRP3 and AIM2 were positively correlated with GBP5 in COAD. Overall, we have demonstrated in multiple ways that GBP5 plays a role in TME of multiple cancers.

### Immunogenicity of GBP5

**FIGURE 5:**
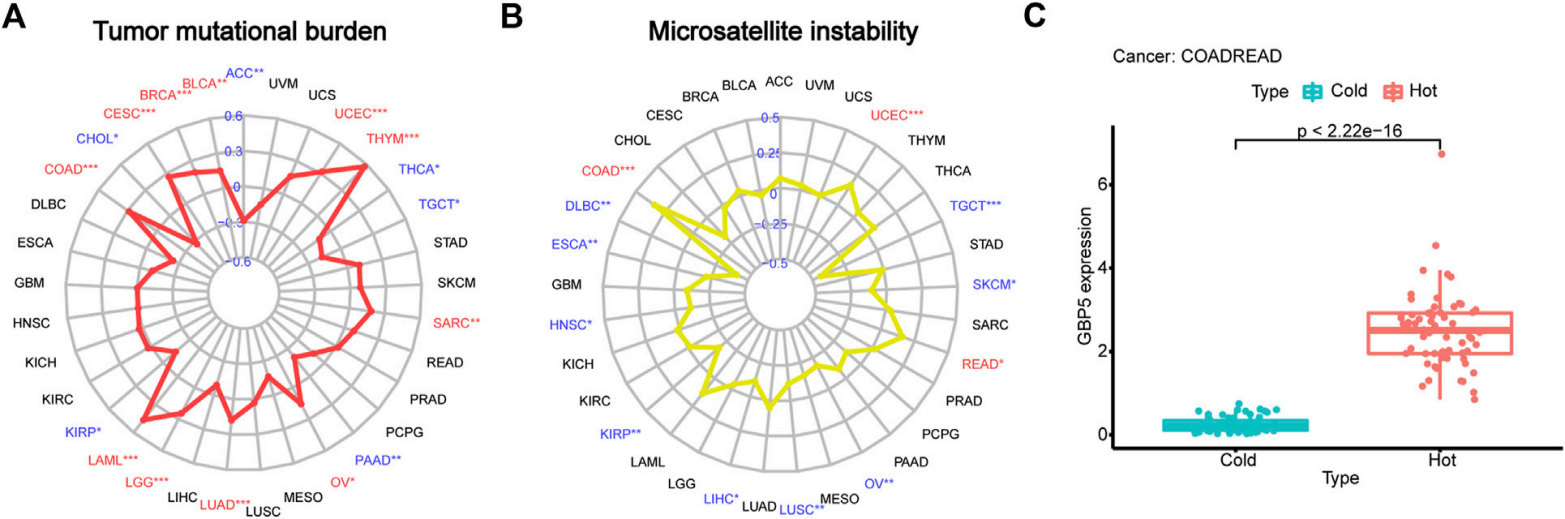
Explanation of GBP5 immunogenicity **(A)** Correlation between GBP5 and TMB (tumor mutation burden) in pan-cancer. Respectively, the correlation number and *p*-value are 0.15 and 0.0024 in BLCA (Bladder urothelial carcinoma), 0.2 and 5.3e-10 in BRCA (Breast invasive carcinoma), 0.27 and 5.2e-06 in CESC (Cervical squamous cell carcinoma and endocervical adenocarcinoma), 0.28 and 8.3e-09 in COAD, 0.46 and 0.00012 in LAML (Acute myeloid leukemia), 0.24 and 3.7e-08 in LGG (Brain lower grade glioma), 0.18 and 4.9e-05 in LUAD (Lung adenocarcinoma), 0.16 and 0.01 in OV (Ovarian serous cystadenocarcinoma), 0.18 and 0.0045 in SARC (Sarcoma), 0.57 and 1.3e-11 in THYM (Thymoma), 0.32 and 1.2e-13 in UCEC (Uterine corpus endometrial carcinoma) **(B)** Correlation between GBP5 and MSI (microsatellite instability) in pan-cancer. Respectively, the correlation number and *p*-value are 0.34 and 5.3e-13 in COAD, 0.16 and 0.049 in READ, 0.17 and 0.00011 in UCEC. **(C)** Differences of GBP5 expression between hot and cold tumors in CRC. The Wilcoxon test is used for the significance test. “*” indicates *p* < 0.05, “**” indicates *p* < 0.01 and “***” indicates *p* < 0.001.

The correlation between GBP5 and TMB or MSI in pan-cancer was compared to better understand the predictive role of GBP5 in ICB response. In COAD and some other types of tumors, GBP5 was significantly positively correlated with TMB and MSI (Figures 5A,B). Given the good clinical and TME association of GBP5 in CRC, we believe that CRC may be a good tumor type for anti-GBP5 therapy. Therefore, we divided CRC samples into hot tumors and cold tumors according to the degree of CD8^+^ T cell infiltration and found that GBP5 was significantly enriched in hot tumors (Figure 5C). These results may partially explain the immunogenicity of GBP5 in CRC.

### Analysis of GBP5 expression in CRC by scRNA-seq data

**FIGURE 6:**
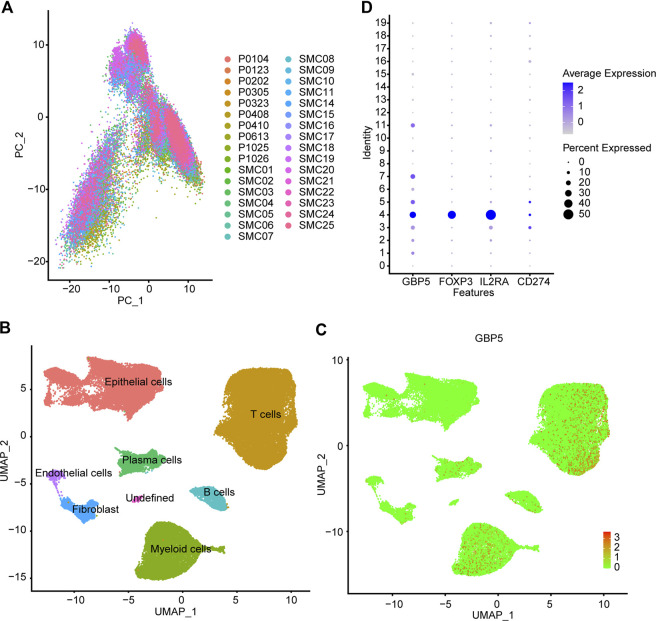
Single cell analysis of GBP5 expression in CRC **(A)** PCA (Principal component analysis) shows no significant separation of CRC samples **(B)** According to the composition of marker genes, the 20 cell clusters are annotated by singleR and CellMarker. **(C)** Expression of GBP5 at single cell level in CRC **(D)** The expression of GBP5, FOXP3, IL2RA and CD274 (PD-L1) in 20 cell clusters.

These results suggested that GBP5 was associated with various clinical and immunological indicators in a variety of tumors, especially CRC. We then focused on the role of GBP5 in CRC. Single-cell RNA-sequencing (scRNA-seq) analysis was performed using CRC patients’ cohorts GSE146771 and GSE132465 to further understand the expression characteristics of GBP5 in immune cells. Firstly, we performed quality control on the scRNA-seq data. PCA (Principal component analysis) showed no significant separation between different CRC samples (Figure 6A). The cells were divided into 20 cell clusters by UMAP algorithm clustering (Supplementary Figures S10, S11). Subsequent annotation results showed that GBP5 was mainly expressed in myeloid cells and a portion of T cells (Figures 6B,C). Interestingly, GBP5 expression in T cells was similar to regulatory T cells (Tregs) markers FOXP3 and IL2RA (Figures 6D, Supplementary Figure S12). Our results suggested that GBP5 has the potential to be biomarkers for certain macrophages or Tregs and may influence anti-tumor immunity through these immune cells.

### Identification and functional analysis of GBP5 related DEGs

**FIGURE 7:**
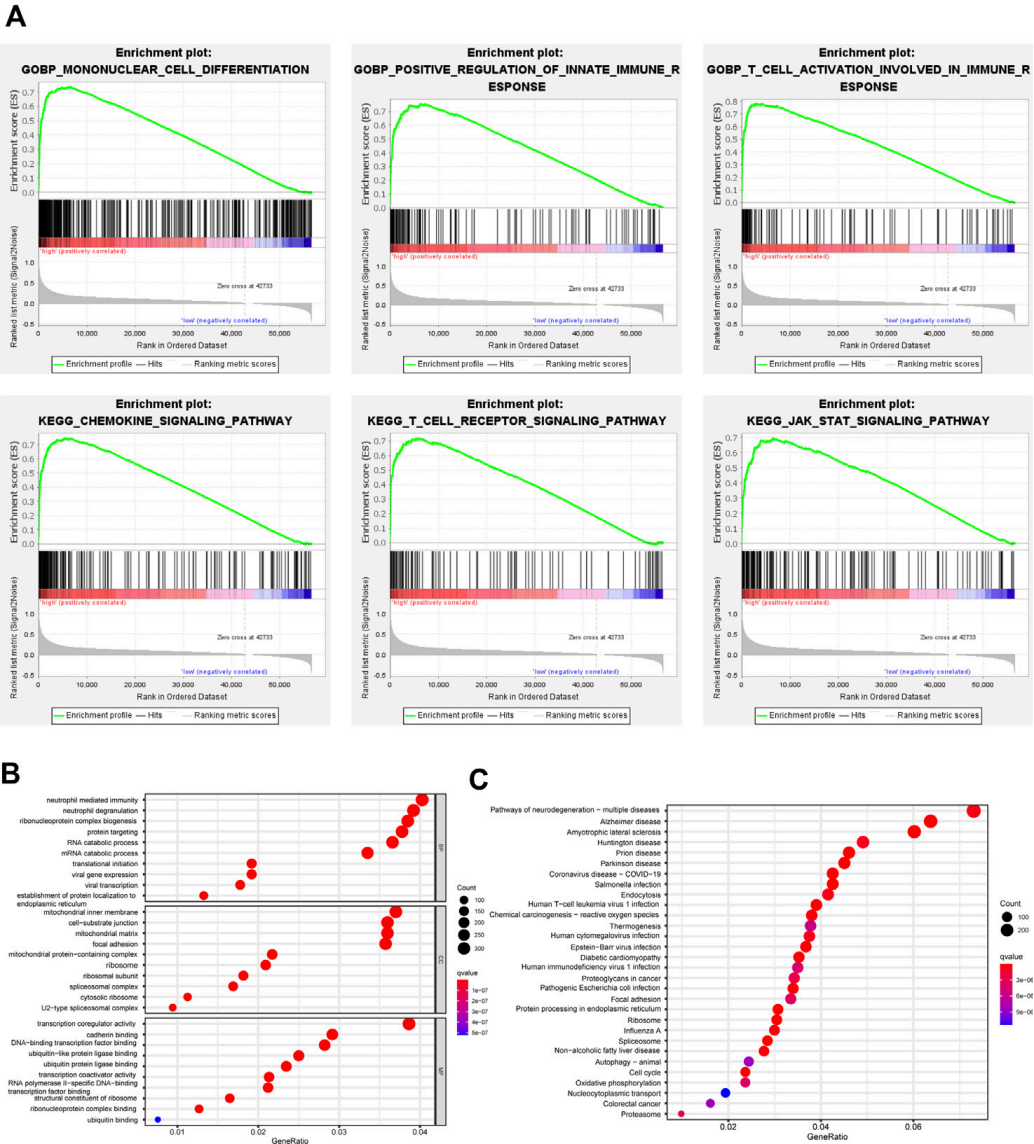
Functional analysis of GBP5 in CRC **(A)** GSEA identifies different gene sets between high and low expression groups of GBP5. The mononuclear cell differentiation (NES = 2.68, p-val = 0.00, FDR q-val = 0.00), the positive regulation of innate immune response (NES = 2.60, p-val = 0.00, FDR q-val = 0.00), the T cell activation involved in immune response (NES = 2.58, p-val = 0.00, FDR q-val = 0.00), the chemokine signaling pathway (NES = 2.53, p-val = 0.00, FDR q-val = 0.00), the T cell receptor signaling pathway (NES = 2.55, p-val = 0.00, FDR q-val = 0.00), and the JAK STAT signaling pathway (NES = 2.53, p-val = 0.00, FDR q-val = 0.00) are enriched in the GBP5 high expression group **(B,C)** GO (Gene Ontology) and KEGG (Kyoto Encyclopedia of Genes and Genomes) analysis of DEGs (differentially expressed genes) between GBP5 high and low expression groups.

Patients in COAD and READ were divided into two groups according to the median value of GBP5 expression and GSEA was performed to identify pathways associated with GBP5. The results showed that the GBP5 high expression group is enriched with many immune-related gene sets, including mononuclear cell differentiation (GO) and chemokine signaling pathway (KEGG) (Figure 7A). Then, we obtained 15,348 DEGs from the GBP5 high and low expression groups (Supplementary Table S2). GO enrichment also demonstrated a strong correlation between the key genes associated with GBP5 and immunity (Figure 7B). For example, GBP5 is associated with neutrophil mediated immunity and neutrophil degranulation. KEGG enrichment showed DEGs is enriched in oxidative phosphorylation and cell cycle, which may reveal the mechanism of GBP5 in cancer (Figure 7C). In addition to colorectal cancer, GBP5 is also associated with coronavirus disease COVID-19 and Pathogenic *Escherichia coli* infection, indicating that GBP5 may play a role in other diseases. Finally, univariate Cox analysis identified 81 key genes affecting prognosis (Supplementary Table S3). Our results verified the strong correlation between GBP5 and TME.

### Development of a prognostic model for CRC

**FIGURE 8:**
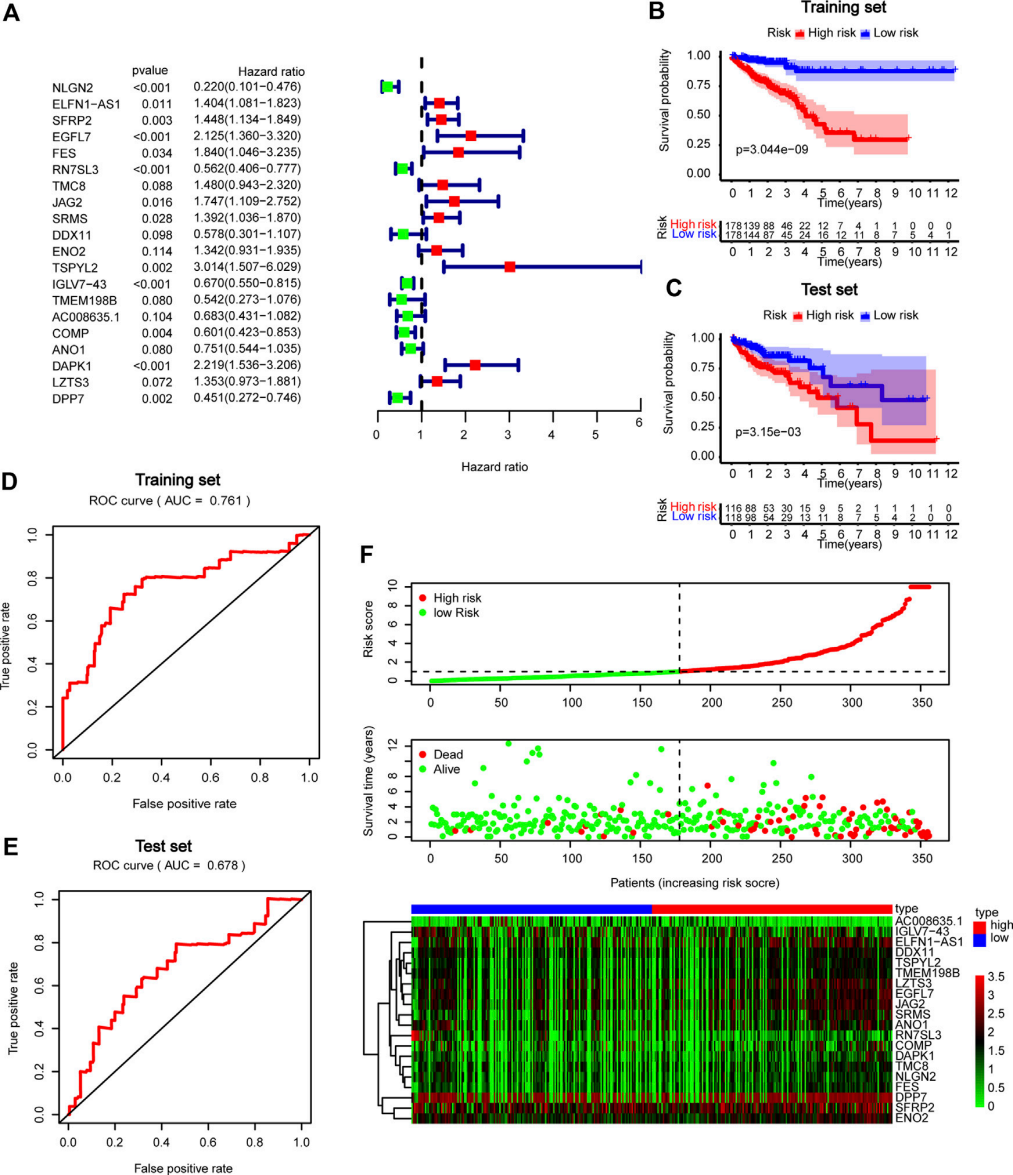
Construction and validation of GBP5 related prognostic models in CRC **(A)** Forest plot in multivariate Cox analysis. **(B,C)** Kaplan-Meier curves of high- and low-risk score groups in training and test sets **(D,E)** ROC curves in training and test sets. **(F)** Risk plot of high- and low-risk score groups.

We further performed multivariate Cox regression analysis in the TCGA training set and obtained 20 genes for developing GBP5 associated risk scores (Figure 8A). Based on these genes, the risk scoring formula was established according to their respective coefficients and expression levels (Supplementary Table S4). Kaplan-Meier survival analysis showed significant statistical differences in both training and test sets based on the same cut-off value (Figures 8B,C). The results showed that patients with high-risk scores had a poor prognosis. The risk score of prediction accuracy verified by the AUC value in the ROC curve was 0.761 and 0.678 in the training set and test set respectively, which was relatively satisfactory (Figures 8D,E). In addition, we can see the correlation between the risk score and the mortality from the survival risk heatmap (Figure 8F). The result showed that patients with a risk score higher than 1.000741 generally had poor survival. The overall mRNA expression levels of DPP7, ENO2, and SFRP2 were higher among the 20 genes, as shown in the heatmap.

### Impairing the proliferation of CRC cells by GBP5

We knocked down or overexpressed GBP5 in the CRC cell line (DLD1) to investigate the effect of GBP5 on the biological behavior of CRC cells. WB results showed that GBP5 was effectively down-regulated or up-regulated at the protein level (Supplementary Figure S13A). Next, EdU assay and colony formation assay were used to evaluate the effect of GBP5 on cell proliferation (Supplementary Figure S13B,C). We found that the cell viability and proliferation ability were significantly reduced when GBP5 was overexpressed, and the results were opposite when GBP5 was knocked down. Therefore, we demonstrated the inhibitory effect of GBP5 on cell proliferation by functional experiments.

## Discussion

We found that GBP5 is elevated in a variety of cancers as a potential immunotherapy target. Previous studies have shown that GBP5 can promote the malignant progression of oral squamous cell carcinoma, triple-negative breast cancer, and glioblastoma (Cheng et al., 2021b; Liu et al., 2021; Yu et al., 2021). However, GBP5 is associated with a good prognosis in many tumors by bioinformatics analysis. Although GBP5 is not strongly correlated with age and sex, GBP5 expression is lower in patients with distant metastasis in COAD and HNSC. In addition, in other gastrointestinal tumors such as STAD and READ, GBP5 also tend to be lower expressed in M1 stage samples, which is consistent with the effect of GBP5 on patient prognosis. Considering the immunogenicity of GBP5 (Li et al., 2021), we hypothesize that GBP5 can affect the prognosis by recruiting some tumor-suppressing immune cells.

Next, we further analyze the relationship between GBP5 and TME. GBP5 has been reported to be associated with tumor immune invasion in basal-like breast cancer and hepatocellular carcinoma (Cimas et al., 2020; Xiang et al., 2021). Furthermore, we found that GBP5 mRNA strongly correlated with immune invasion in almost all tumors. In particular, GBP5 expression had a positive relationship with the proportion of Macrophages M1, which is consistent with previous studies (Fujiwara et al., 2016). Moreover, GBP5 was also closely associated with CD8^+^ T cells. CD8^+^ T cells can not only affect tumor growth, but also be an important indicator of inflamed TME (Hu et al., 2021). GBP5 is positively correlated with the expression of some immune inhibitors such as PD1, PD-L1, and CTLA-4. PD-1 and PD-L1 are immune-checkpoint proteins that interact with each other and can inhibit adaptive antitumor immune response (Doroshow et al., 2021). Immune-checkpoint inhibitors targeting PD-1 or PD-L1 have been shown to improve prognosis in patients with many types of cancer but only in a small number of patients (Gong et al., 2018). Anti-CTLA4 therapy has been applied clinically with serious adverse effects (Zhang et al., 2019b). Other genes in the top ten correlated genes are also immune inhibitors. Upregulation of immune inhibitors is one of the key features of an inflamed TME, which is necessary for the success of ICB (Spranger et al., 2013). However, the correlation between GBP5 and immune inhibitors and its clinical significance needs further functional experimental confirmation. In addition, GBP5 has the potential to predict ICB response, which provides a broader possibility for clinical application of GBP5. In COAD and UCEC, GBP5 is positively correlated with TMB and MSI, both of which have the potential to predict immunotherapy effects (Chan et al., 2019; Yamamoto and Imai, 2019). Similarly, GBP5 was highly expressed in hot tumors, suggesting that GBP5 may define inflammatory TME. Therefore, GBP5 is an immunogenicity gene and CRC can be a suitable candidate for targeting GBP5.

scRNA-seq is an emerging single-cell analysis assay that characterizes tumor-infiltrating immune cells (Zhang and Zhang, 2020). By analyzing the scRNA-seq of CRC samples, we found GBP5 elevated expression levels in immune cells, especially myeloid cells and T cells. The results of the single-cell analysis are consistent with the above results of bulk RNA-seq. In particular, GBP5 and some Treg markers, such as FOXP3 and IL2RA, are specifically expressed in cluster 4, indicating that GBP5 also has the potential to become Treg markers. In tumors, however, Tregs play a role in suppressing anti-tumor immunity (Togashi et al., 2019). In addition, the relationship between Tregs and immunotherapy remains unclear. In general, the relationship between GBP5 and Tregs needs further study.

In function, GBP5 is also associated with tumor immunity, which we demonstrated using a variety of methods, including GSEA enrichment analysis. In addition to the functions or pathways related to macrophage and T cell, chemokine signaling pathway and JAK-STAT signaling pathway are also enriched in high GBP5 groups, which may be worth further study in the future. Among the 20 genes we finally obtained for constructing GBP5 related prognostic models, ELFN1-AS1 is a long non-coding RNA (LncRNA). In some cancers, ELFN1-AS1 has been verified to promote the proliferation and migration of cancer cells. In addition, lncRNA ELFN1-AS1 also acts as a sponge of miRNA (Jie et al., 2020; Lei et al., 2020; Zhang et al., 2020). TMC8 is a member of Transmembrane channel-like (TMC) protein. Recent studies have shown that TMC8 has the potential to be a biomarker of immunotherapy response and prognosis (Song et al., 2021). TSPYL2 inhibits tumor growth and is thought to be associated with DNA damage (Magni et al., 2019). Some genes in the prognostic model have been well studied. For example, SFRP2 methylation has been an important indicator of CRC detection and diagnosis for a long time (Zhang et al., 2015). In parallel, the significance of other genes such as NLGN2, RN7SL3, and IGLV7−43 in tumors is unclear. Thus, we can find valuable genes for further study through the construction of prognostic models. Finally, we demonstrated the inhibitory effect of GBP5 on CRC cell proliferation by establishing an *in vitro* model. However, more studies are needed to clarify the relationship between GBP5 and immunotherapy. Additionally, other members of GBPs including GBP3 and GBP4 also need further research.

This is the first bioinformatics study of GBP5 in pan-cancer and the first analysis of GBP5 using scRNA-seq. Our study is very useful for the application of GBP5 in immunotherapy. Although this is a preliminary result, it lays the foundation for the future study of GBP5 in cancer.

## Conclusion

This study mainly explores the correlation between GBP5 and TME. We have reason to believe that our findings will be helpful for future research and the application of GBP5.

## Data Availability

Publicly available datasets were analyzed in this study. The names of the repository/repositories and accession number(s) can be found in the article/Supplementary Material.
