# Supplementary material for: Critical role of guanylate binding protein 5 in tumor immune microenvironment and predictive value of immunotherapy response
Source: Front Genet. 2022 Sep 30;13:984615. doi: 10.3389/fgene.2022.984615 (PMC9561824; doi:10.3389/fgene.2022.984615)
Supplement: Supplementary file 5 [file DataSheet1.docx]

Supplementary Material

## Supplementary Figures


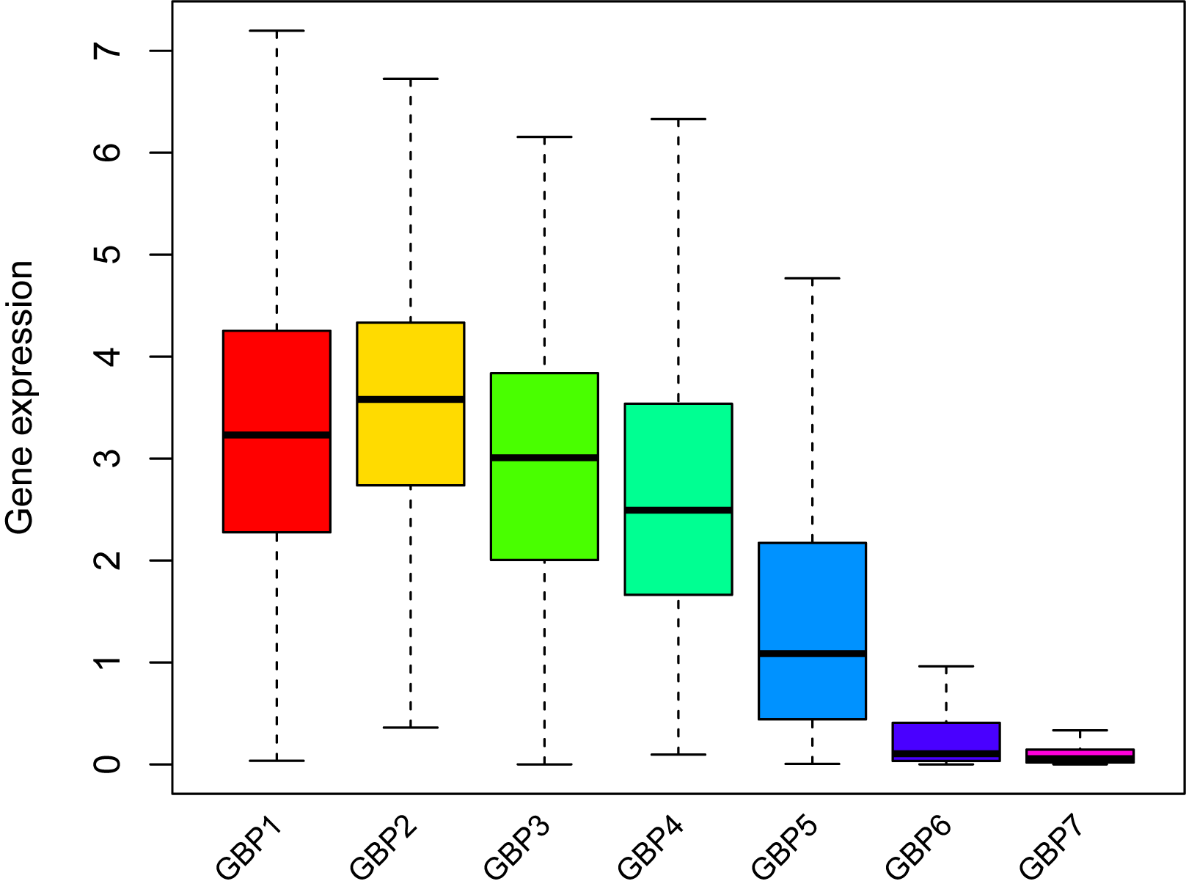


**Supplementary Figure 1.** GBPs mRNA expression in pan-cancer. GBP1 expression is 3.3052845 ± 1.4346234. GBP2 expression is 3.5060576 ± 1.2067510. GBP3 expression is 2.9047787 ± 1.2472609. GBP4 expression is 2.6855462 ± 1.3347150. GBP5 expression is 1.4703770 ± 1.3072968. GBP6 expression is 0.7836507 ± 1.6004056. GBP7 expression is 0.2085625 ± 0.6152793.


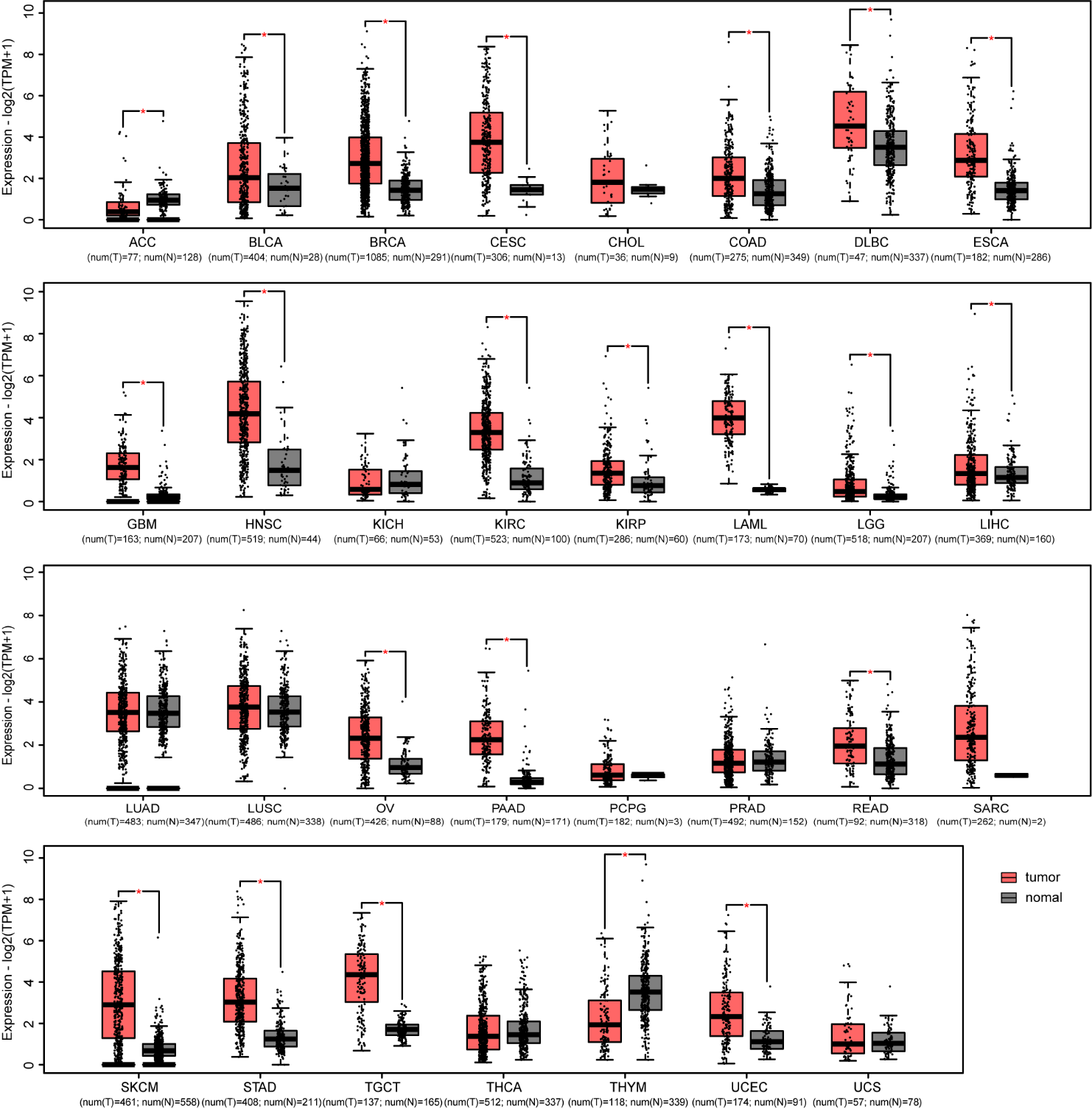


**Supplementary Figure 2.** GBP5 expression difference between tumor and normal tissues in pan-cancer by GEPIA2. Using one-way ANOVA, GBP5 expression is higher in tumor tissue than in normal tissue during most cancer types (22/31). “*” indicates p < 0.05.


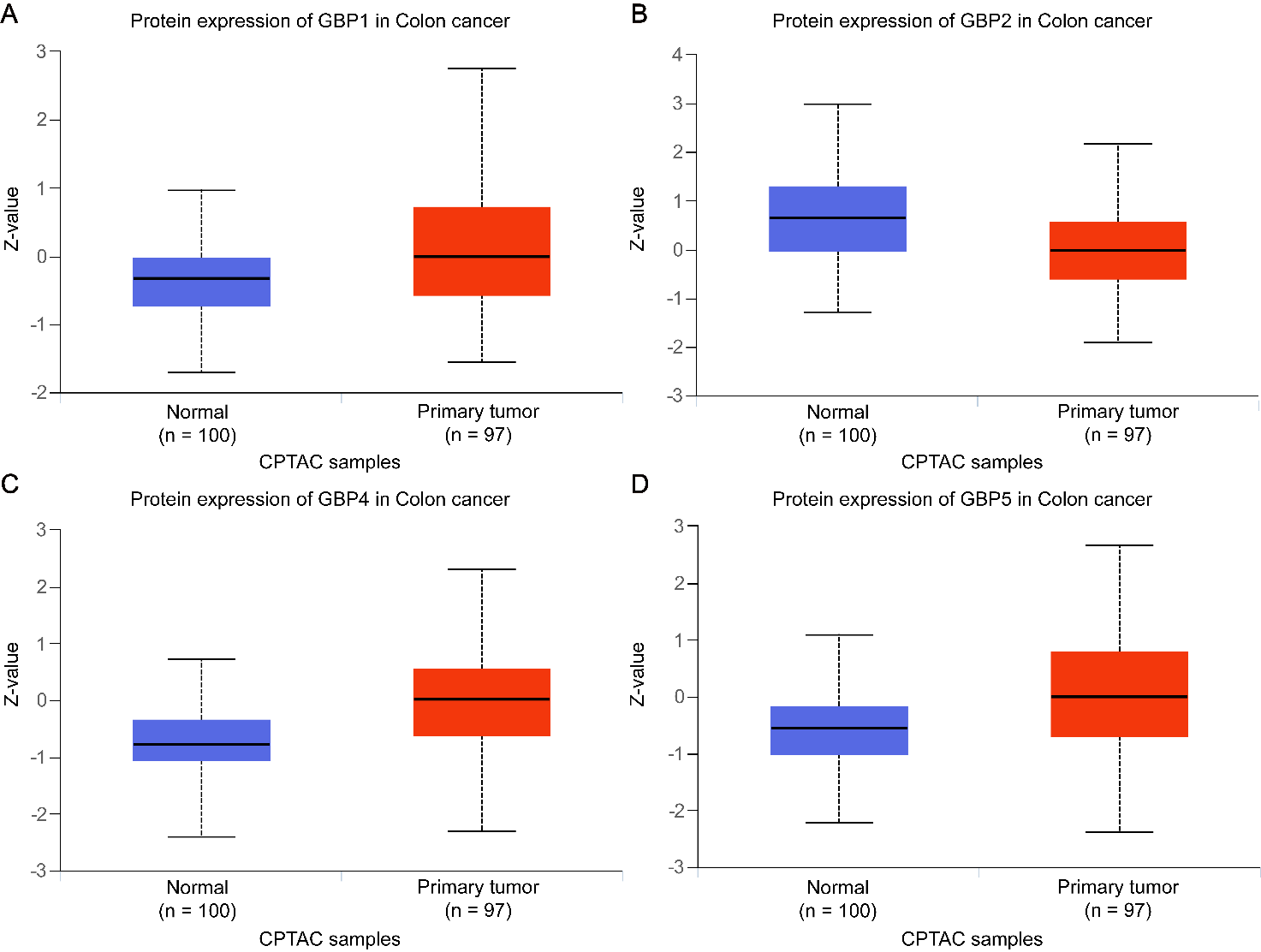


**Supplementary Figure 3.** GBPs expression difference between tumor and normal tissues in Colon cancer by UALCAN. **(A)** GBP1 expression is higher in tumors than in normal tissues (p = 1.460226e-04). **(B)** GBP2 expression is higher in normal tissues than in tissues (p = 1.877130e-04). **(C)** GBP4 expression is higher in tumors than in normal tissues (p = 1.25852508986603e-08). **(D)** GBP5 expression is higher in tumors than in normal tissues (p = 5.20438594686653e-07).


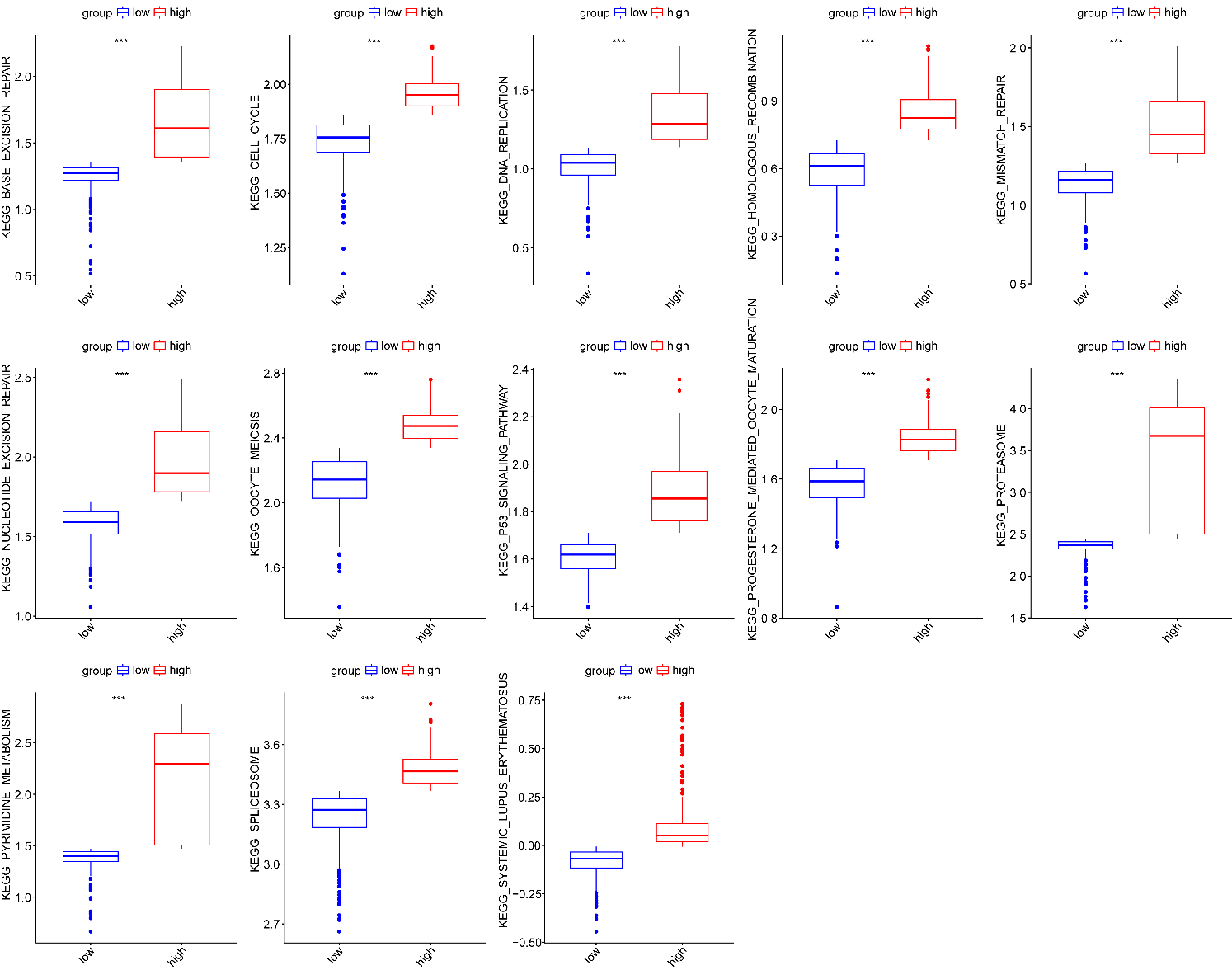


**Supplementary Figure 4.** Differences in enrichment scores of immunotherapy-positive pathways between high and low GBP5 groups in CRC.
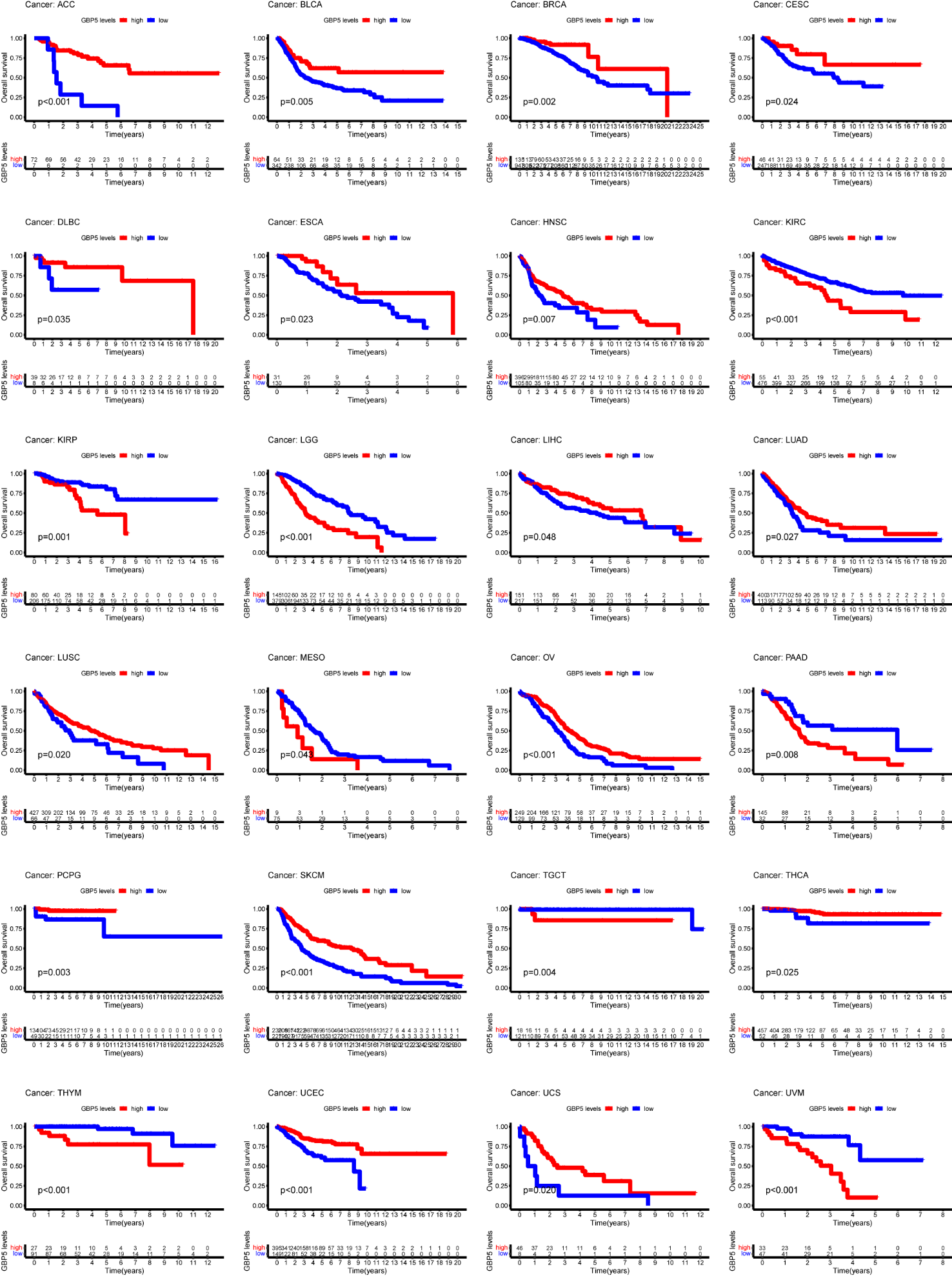


**Supplementary Figure 5.** Kaplan-Meier curves of GBP5 high and low expression groups in pan-cancer. GBP5 is associated with a better short-term prognosis in a majority of cancers (19/27).


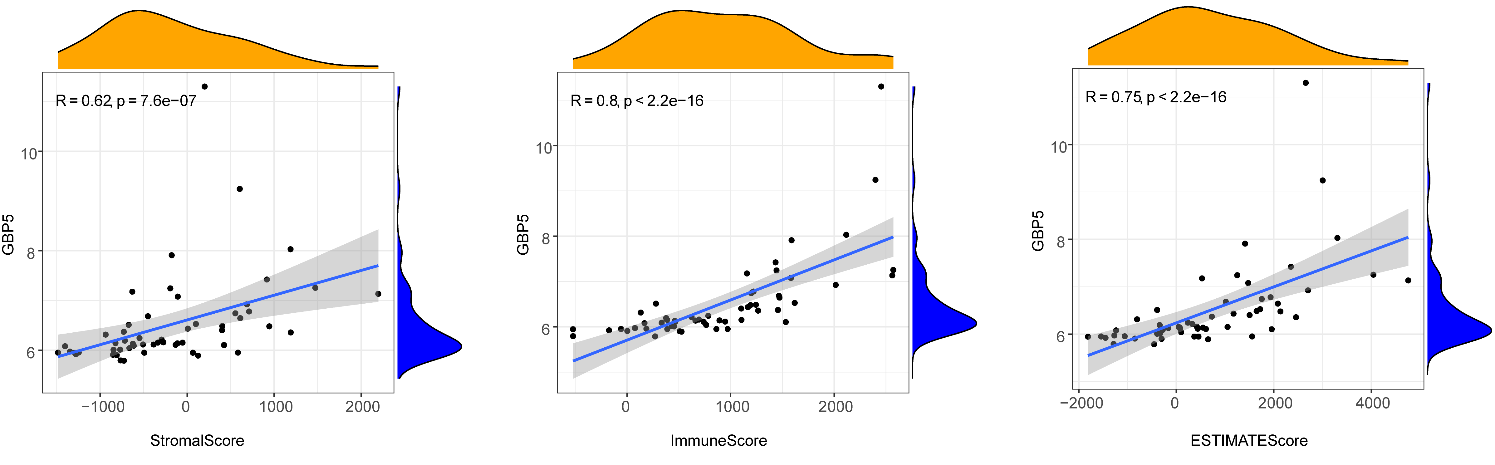


**Supplementary Figure 6.** Correlation between GBP5 and ESTIMATE score in GSE17537.


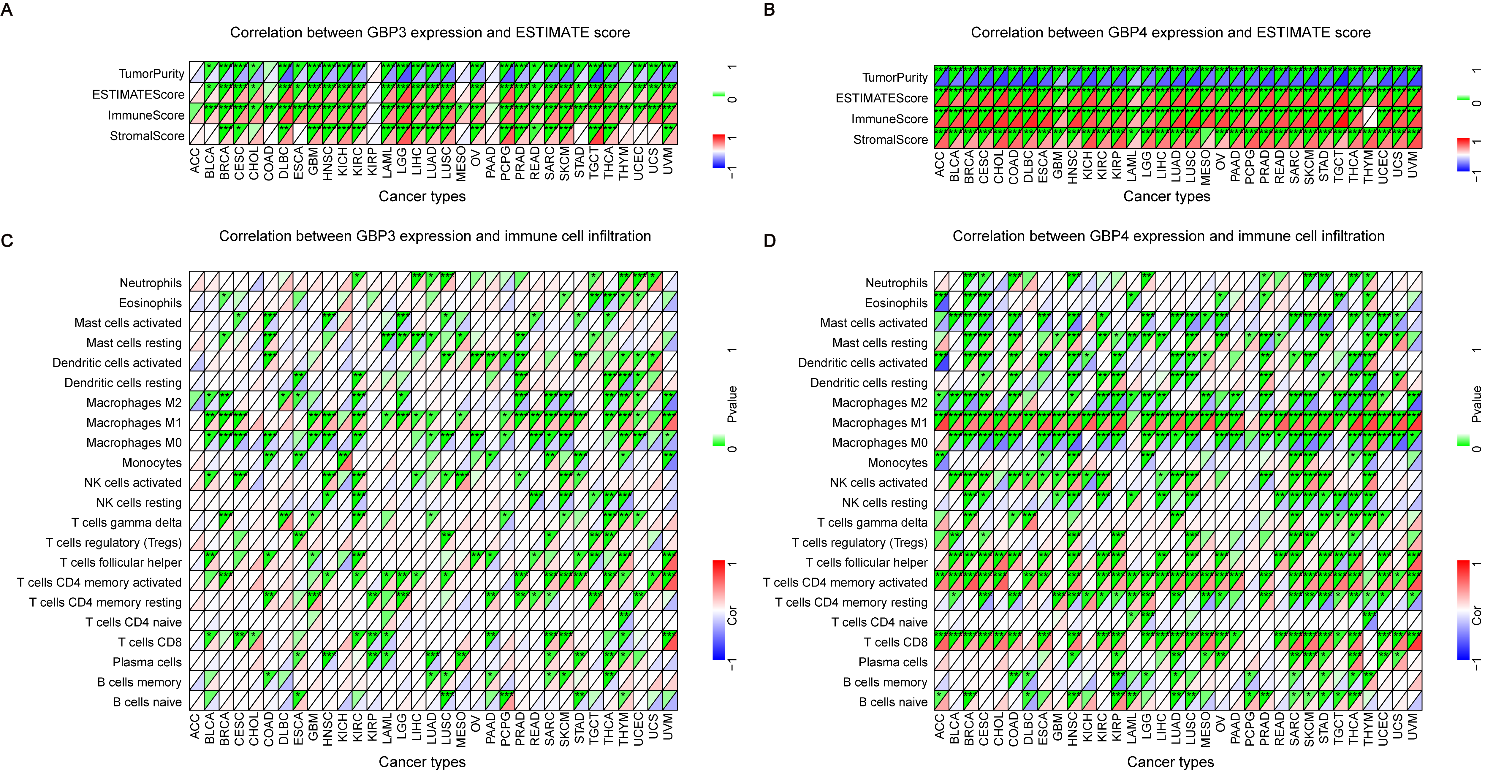


**Supplementary Figure 7.** Correlation between GBP3/4 and TME in pan-cancer. **(A-B)** Correlation between GBP3/4 and ESTIMATE score. **(C-D)** Correlation between GBP3/4 and the infiltration level of TIICs by CIBERSORT.


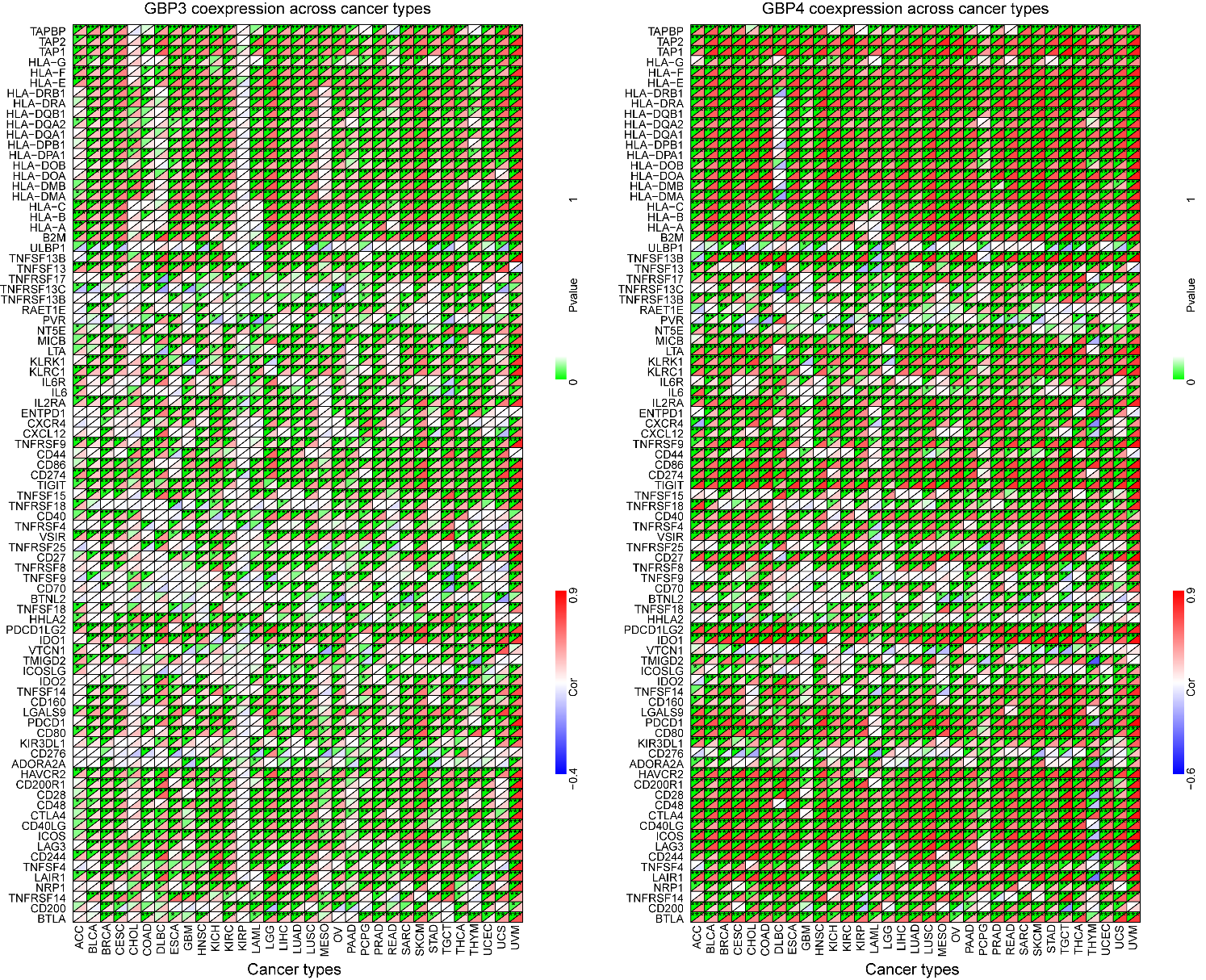


**Supplementary Figure 8.** The expression correlation between GBP3/4 and immune-related genes, including immune inhibitors, immune stimulators, and MHC.


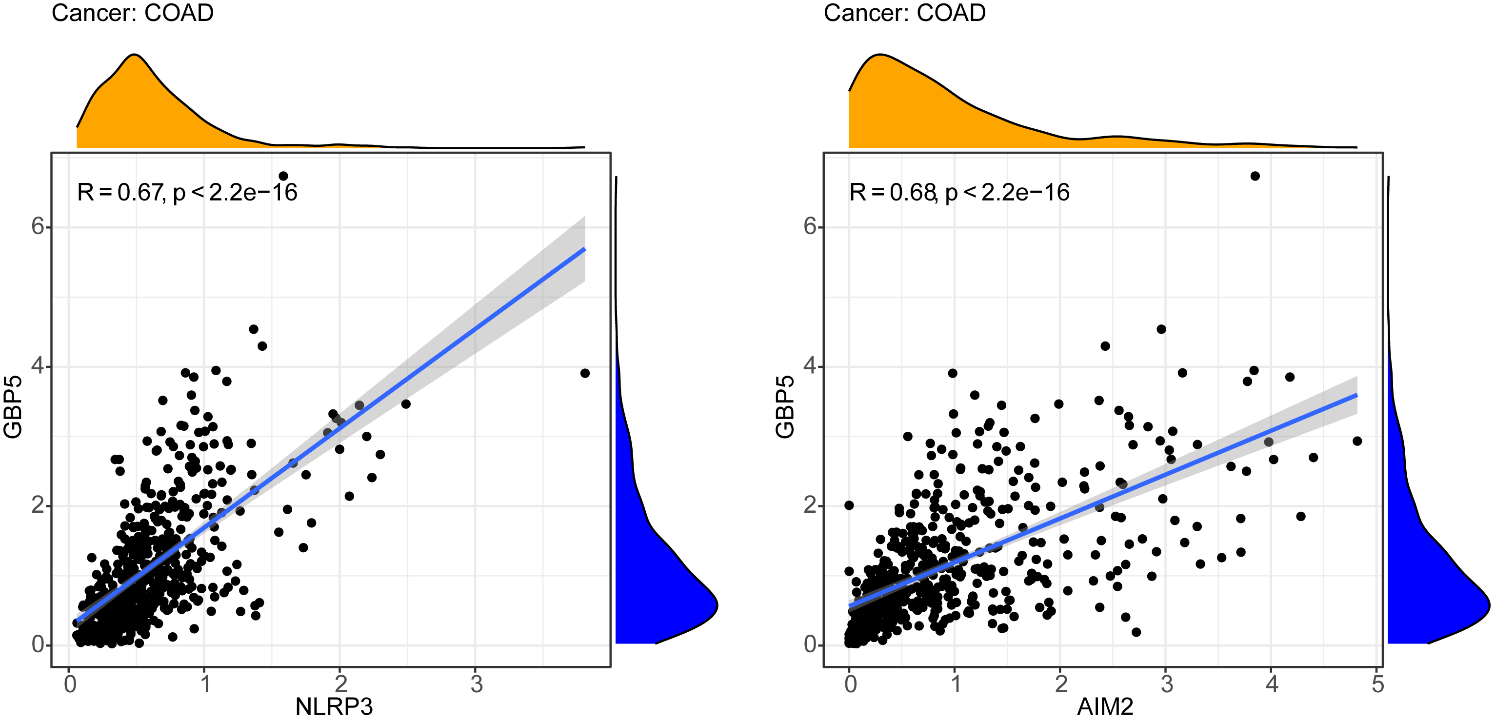


**Supplementary Figure 9.** The expression correlation between GBP5 and inflammasome NLRP3 or AIM2.


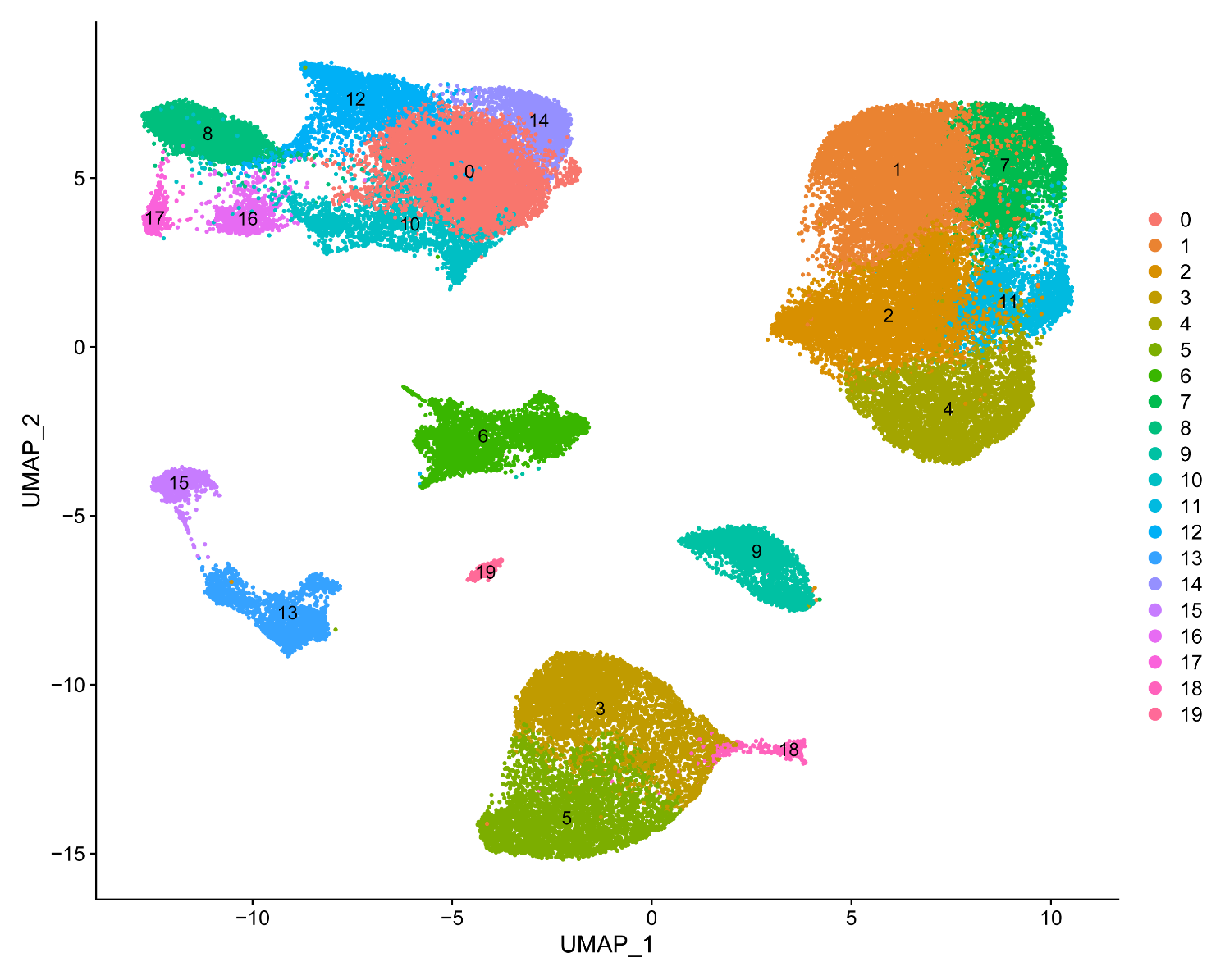


**Supplementary Figure 10.** CRC cells are divided into 20 cell clusters by the UMAP algorithm.


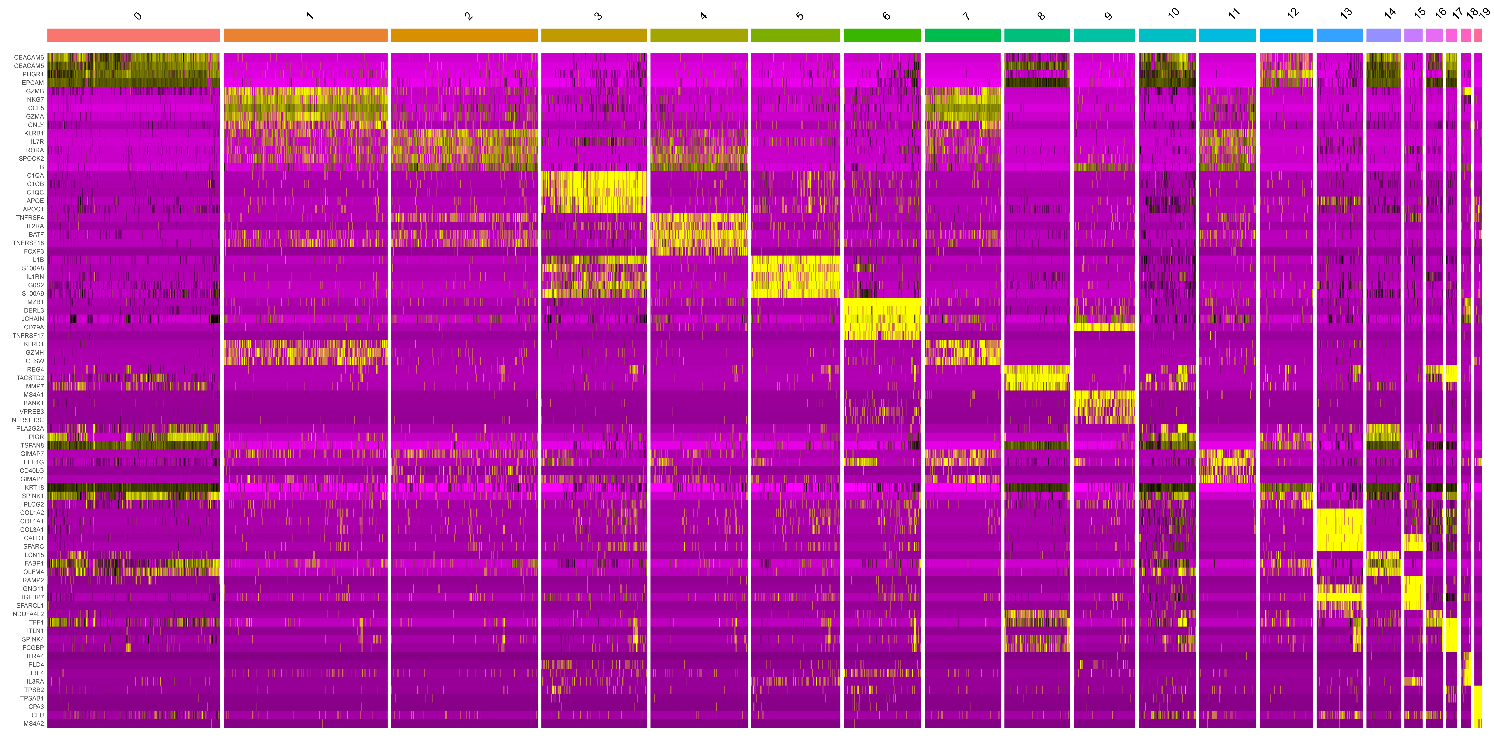


**Supplementary Figure 11.** The heatmap shows the top five marker genes in each cell cluster. The colors from purple to yellow indicate the gene expression levels from low to high.


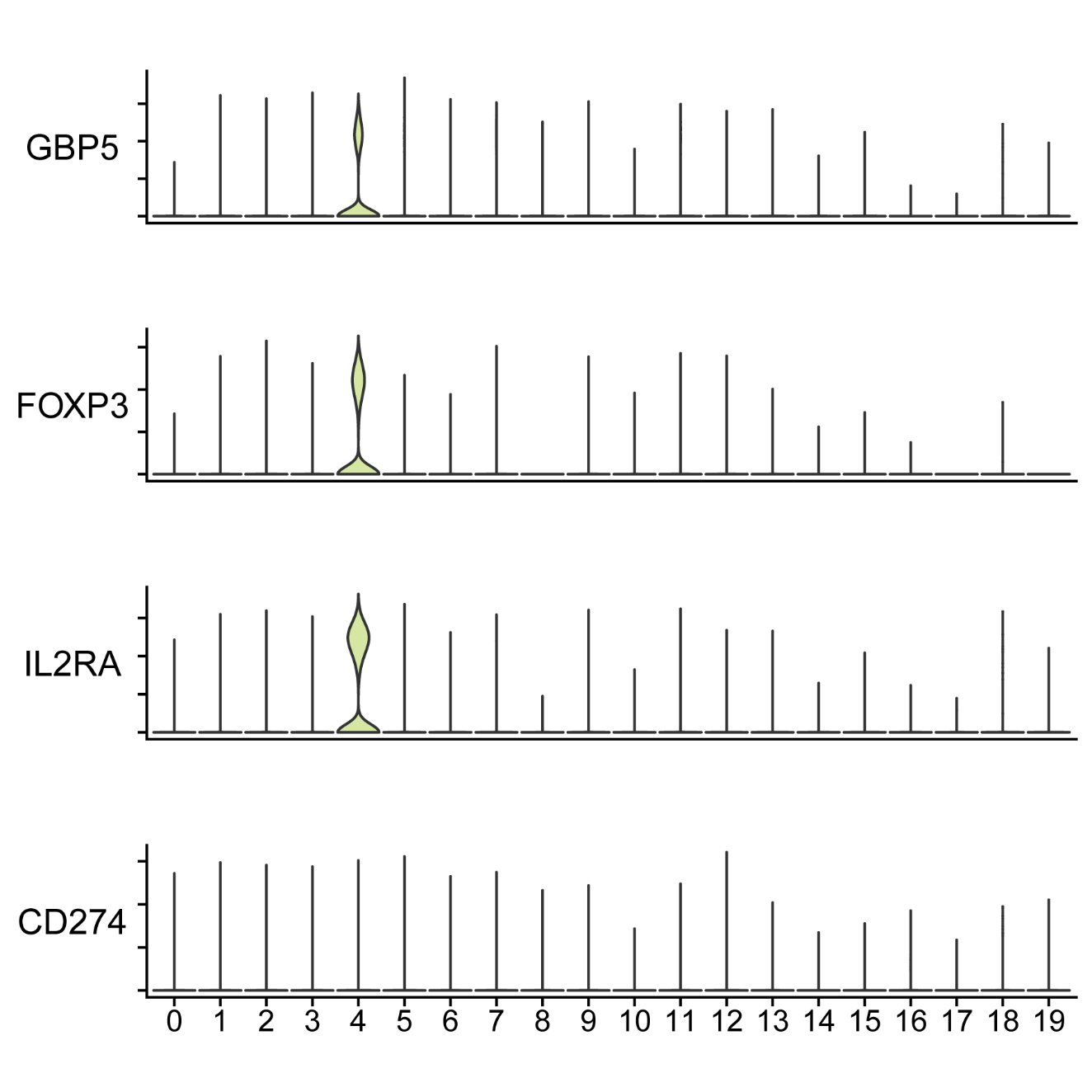


**Supplementary Figure 12.** The violin plot shows the expression of GBP5, FOXP3, IL2RA, and CD274 (PD-L1) in 20 cell clusters. GBP5 expression in cluster 4 is similar to regulatory T cells markers FOXP3 and IL2RA.


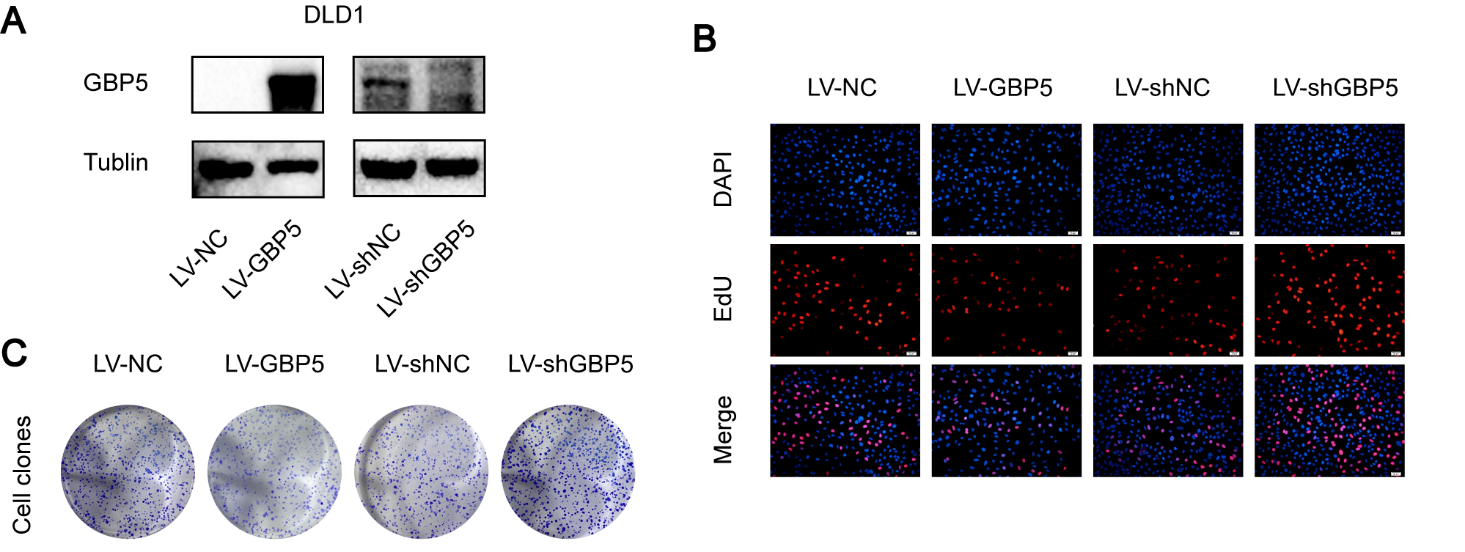


**Supplementary Figure 13.** GBP5 impairs the viability and proliferation of CRC cells. **(A)** Upregulation or downregulation of GBP5 expression in DLD1 cells was confirmed by western blot. **(B)** The viability of GBP5 knockdown or overexpression cells was tested by EdU assay. **(C)** Colony formation assays were performed to assess the effect of GBP5 knockdown or overexpression on the proliferative capacity of cells.
